# Supplementary figures and images for: Concomitant activation of GLI1 and Notch1 contributes to racial disparity of human triple negative breast cancer progression
Source: eLife. 2021 Dec 10;10:e70729. doi: 10.7554/eLife.70729 (PMC8664295; doi:10.7554/eLife.70729)

## Slide 1
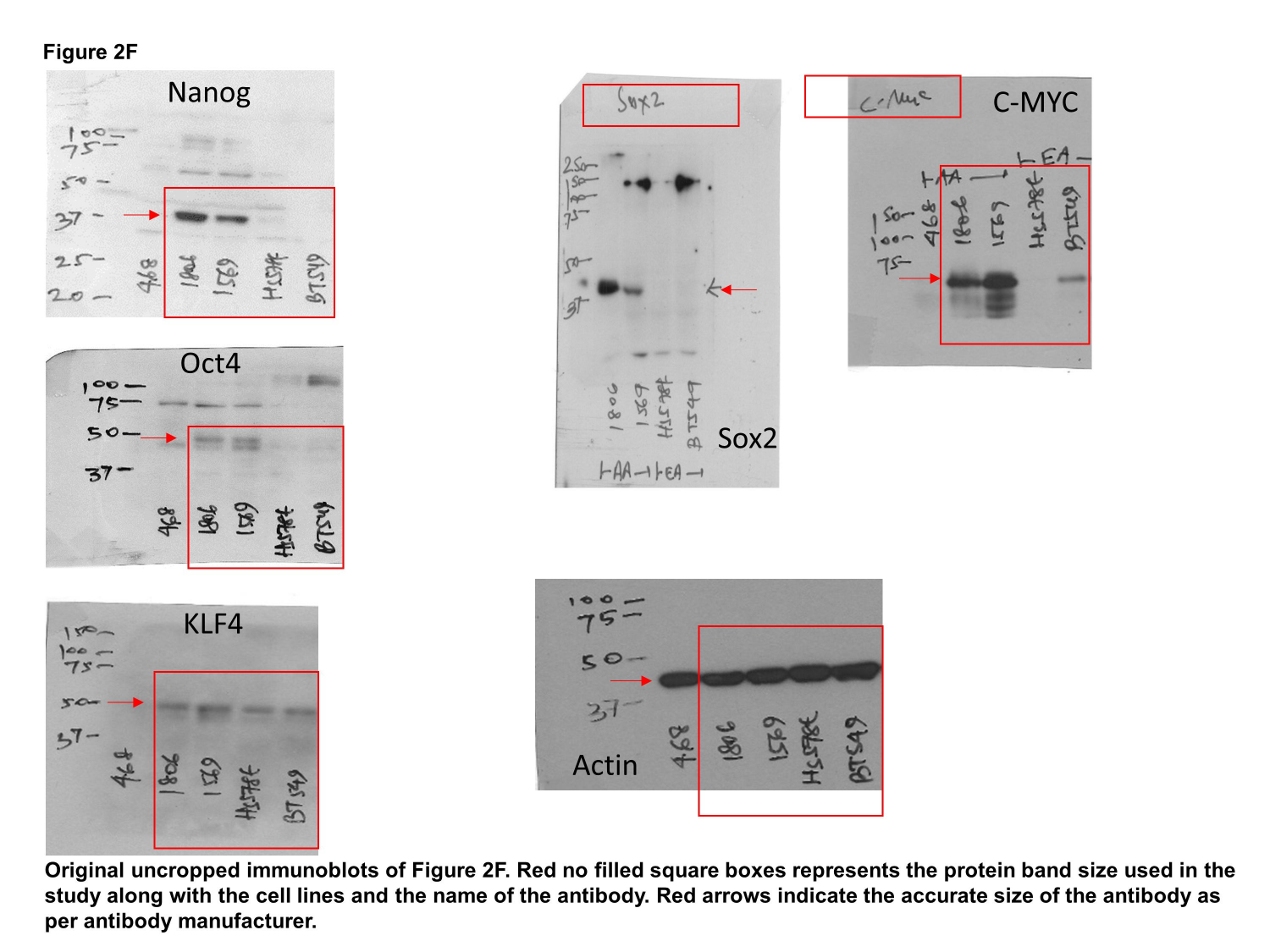

Supplement: Figure 2—source data 1. [file elife-70729-fig2-data1.pptx]

## Slide 1
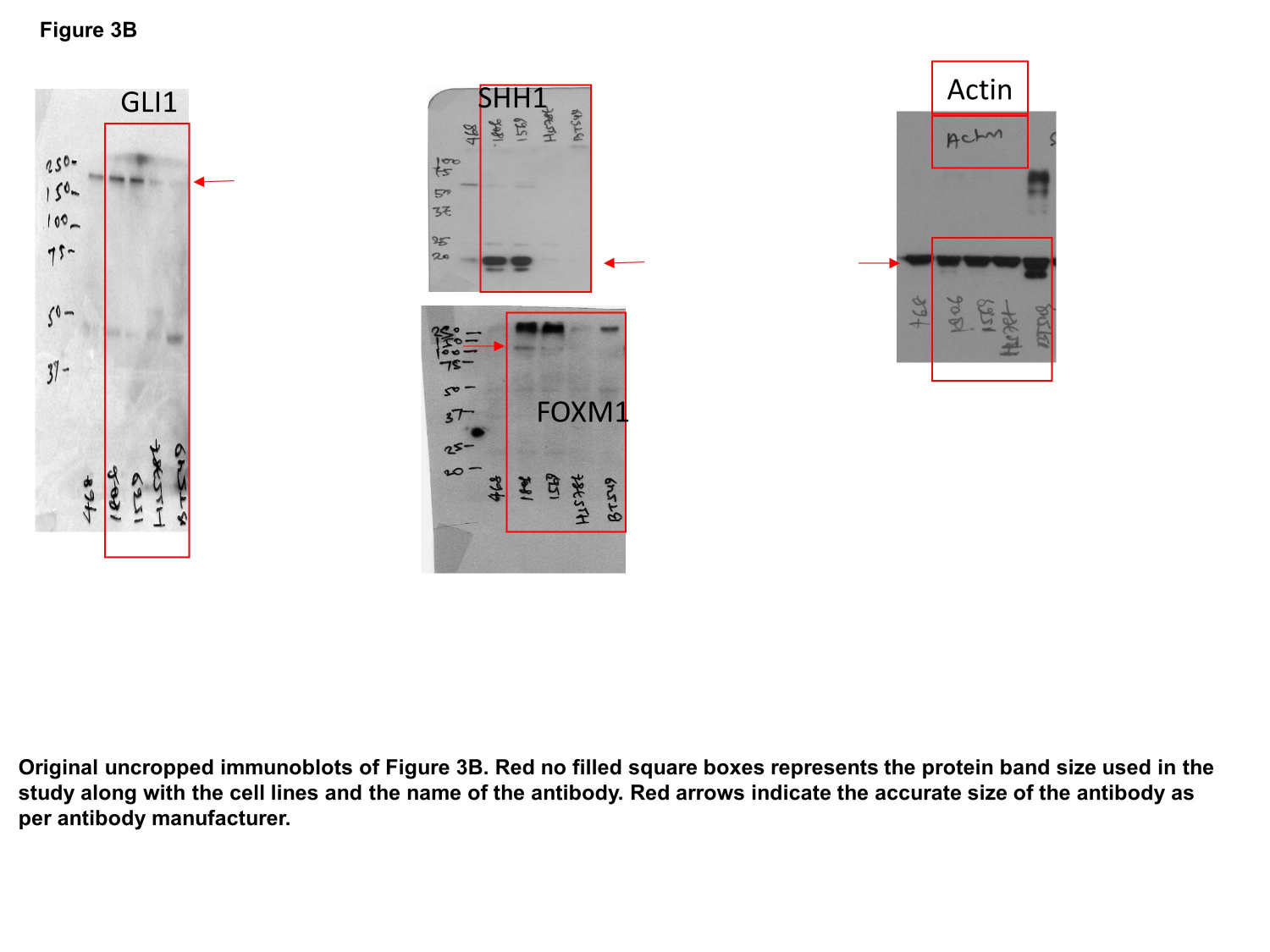

Supplement: Figure 3—source data 1. [file elife-70729-fig3-data1.pptx]

## Slide 1
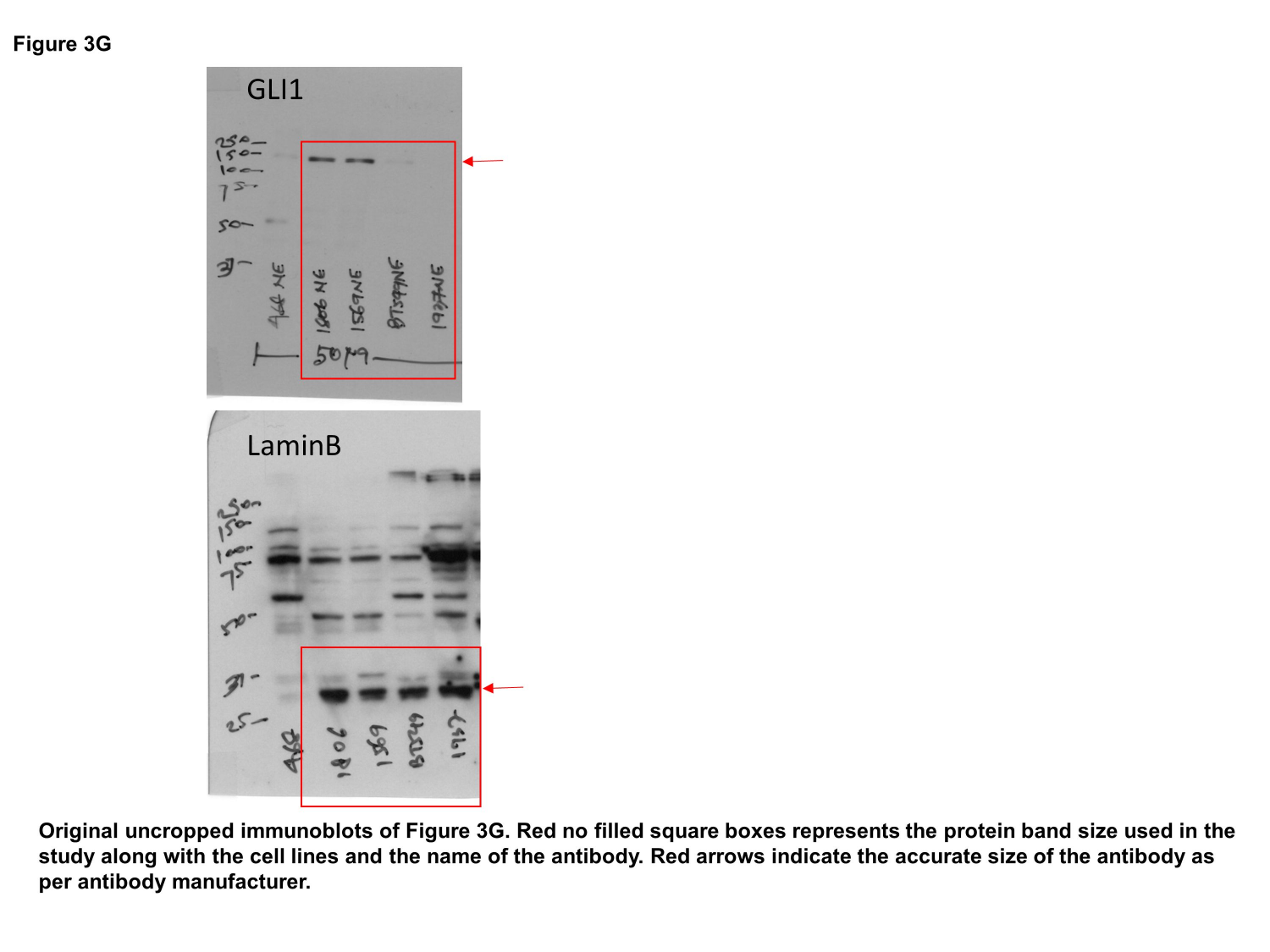

Supplement: Figure 3—source data 2. [file elife-70729-fig3-data2.pptx]

## Slide 1
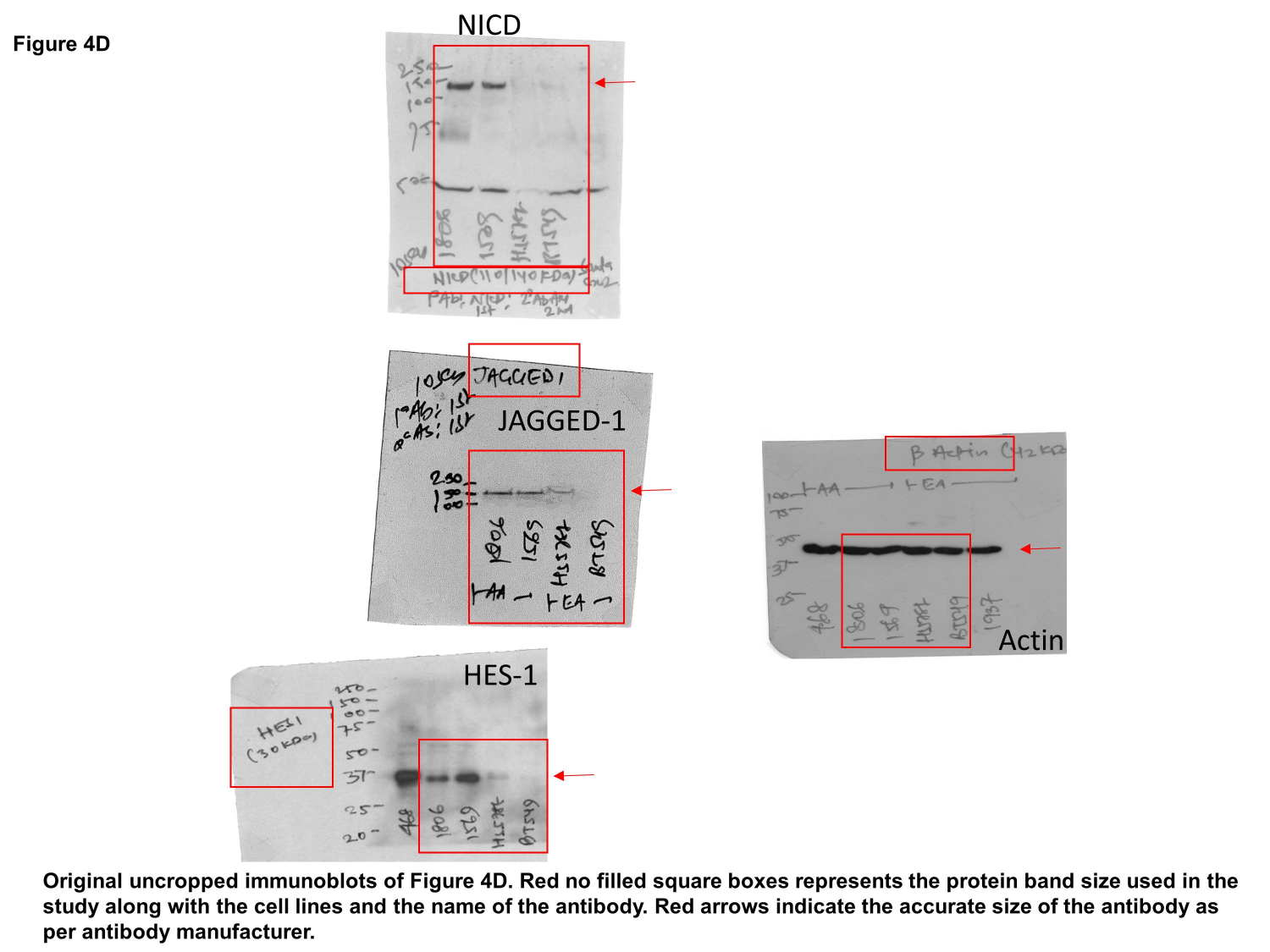

Supplement: Figure 4—source data 1. [file elife-70729-fig4-data1.pptx]

## Slide 1
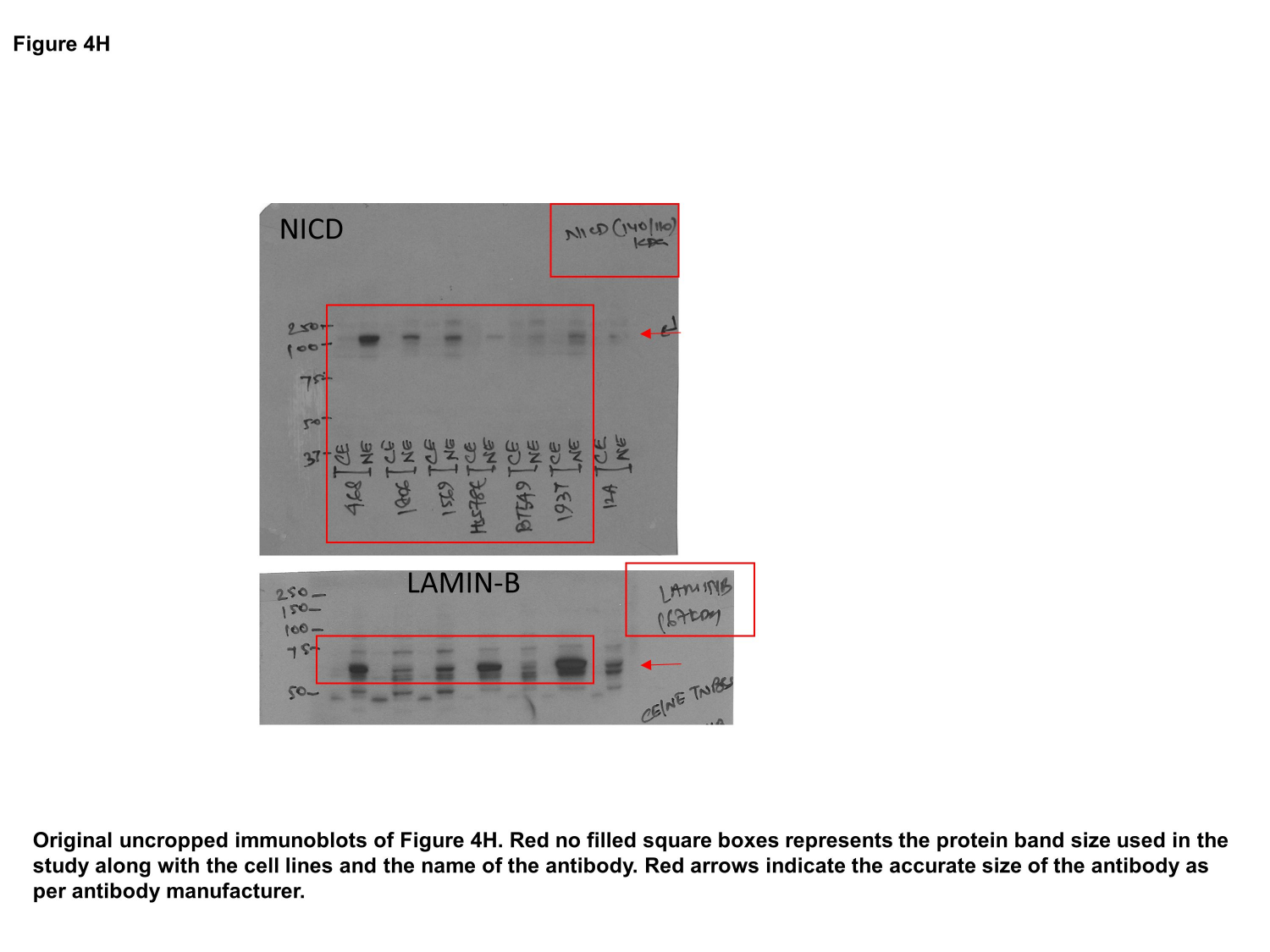

Supplement: Figure 4—source data 2. [file elife-70729-fig4-data2.pptx]

## Slide 1
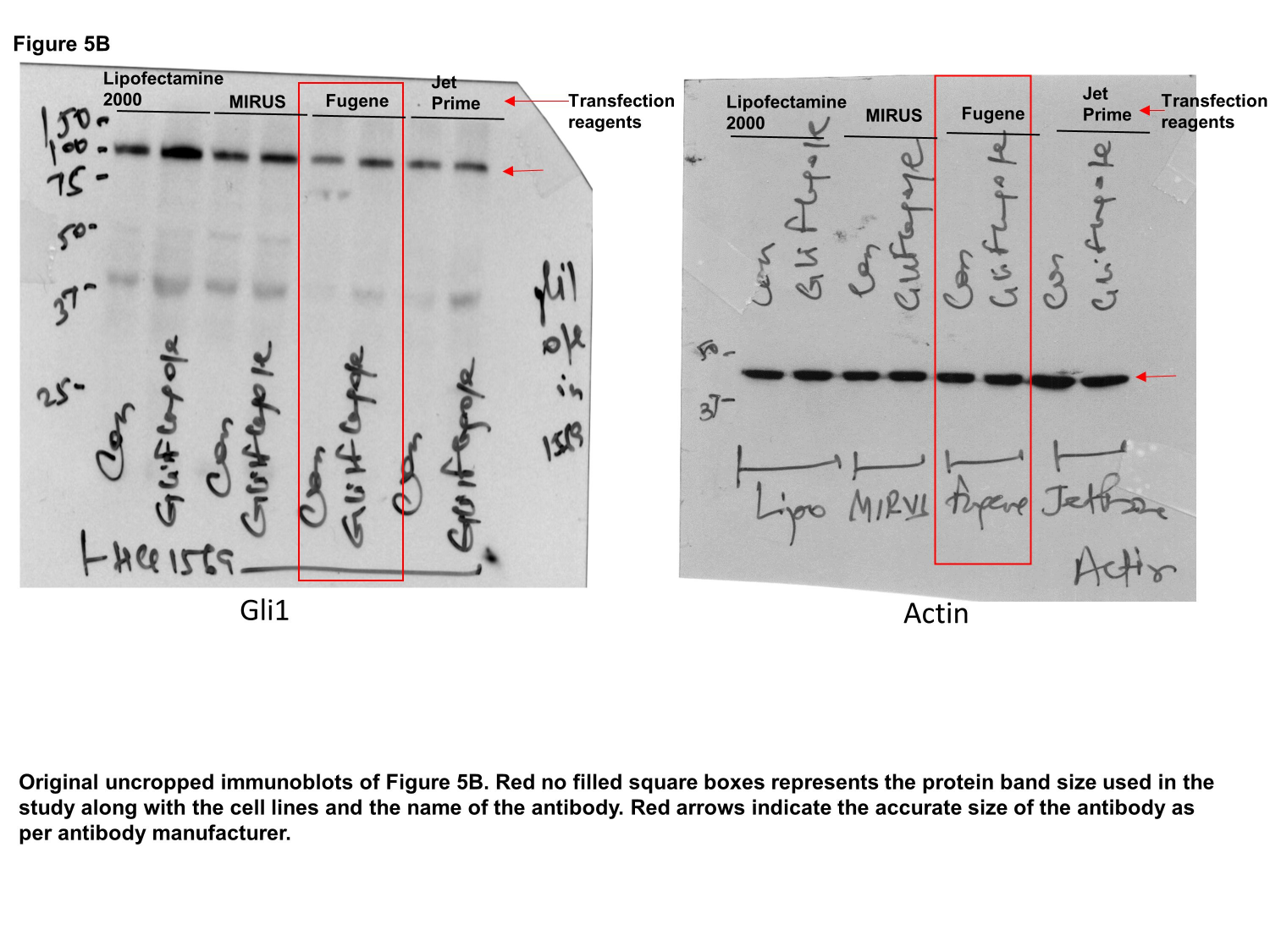

Supplement: Figure 5—source data 1. [file elife-70729-fig5-data1.pptx]

## Slide 1
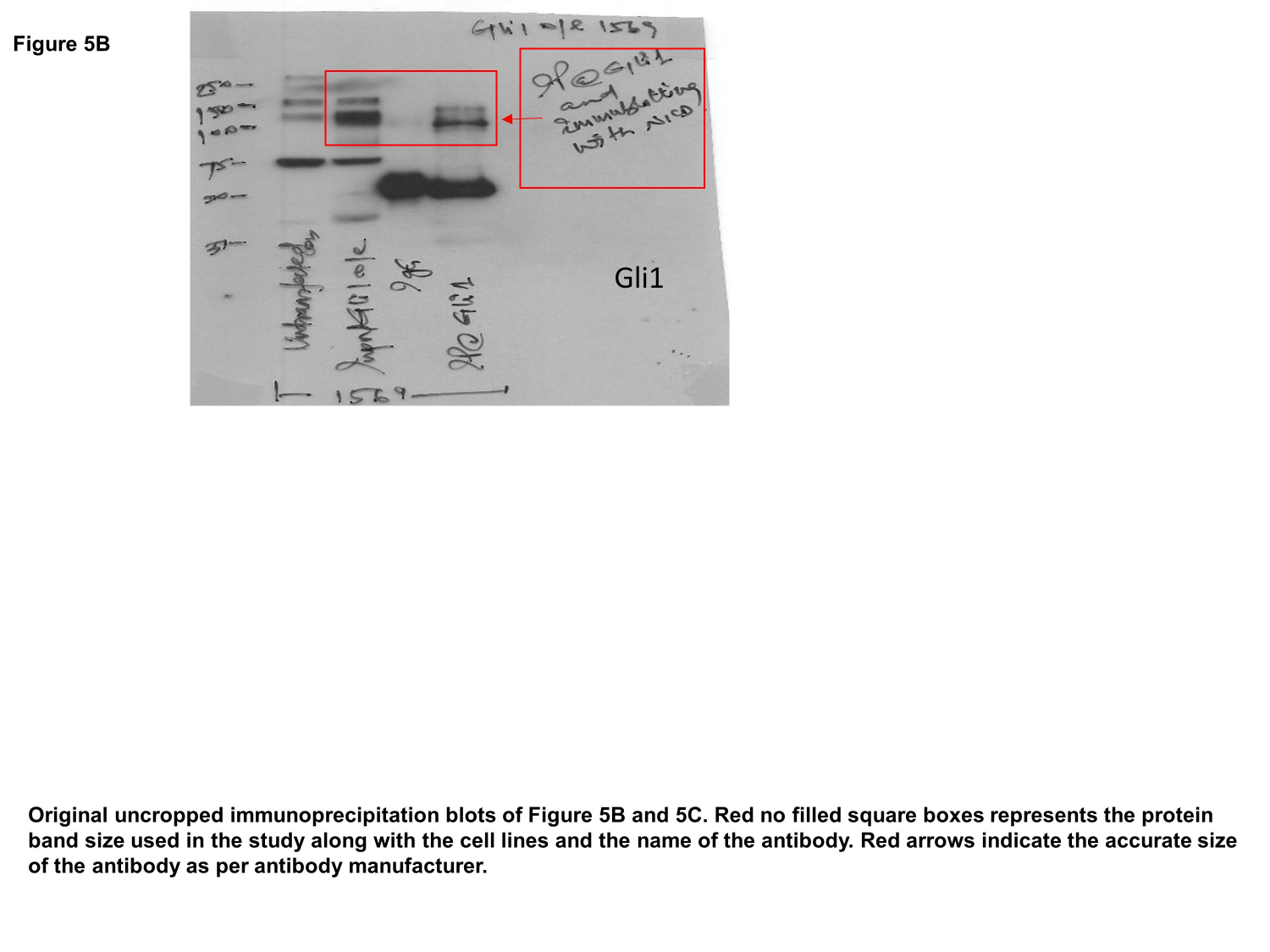

Supplement: Figure 5—source data 2. [file elife-70729-fig5-data2.pptx]

## Slide 1
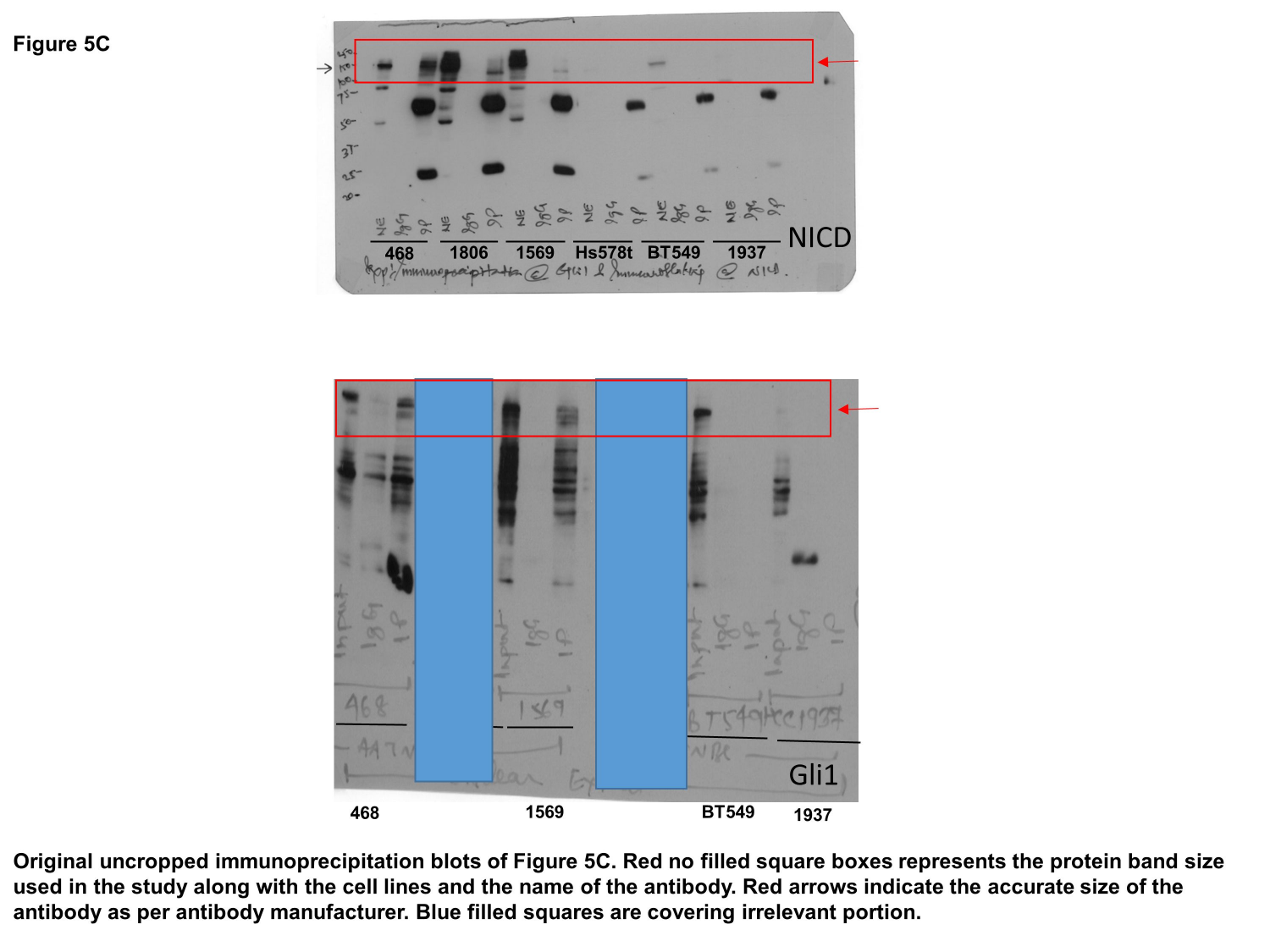

Supplement: Figure 5—source data 3. [file elife-70729-fig5-data3.pptx]

## Slide 1
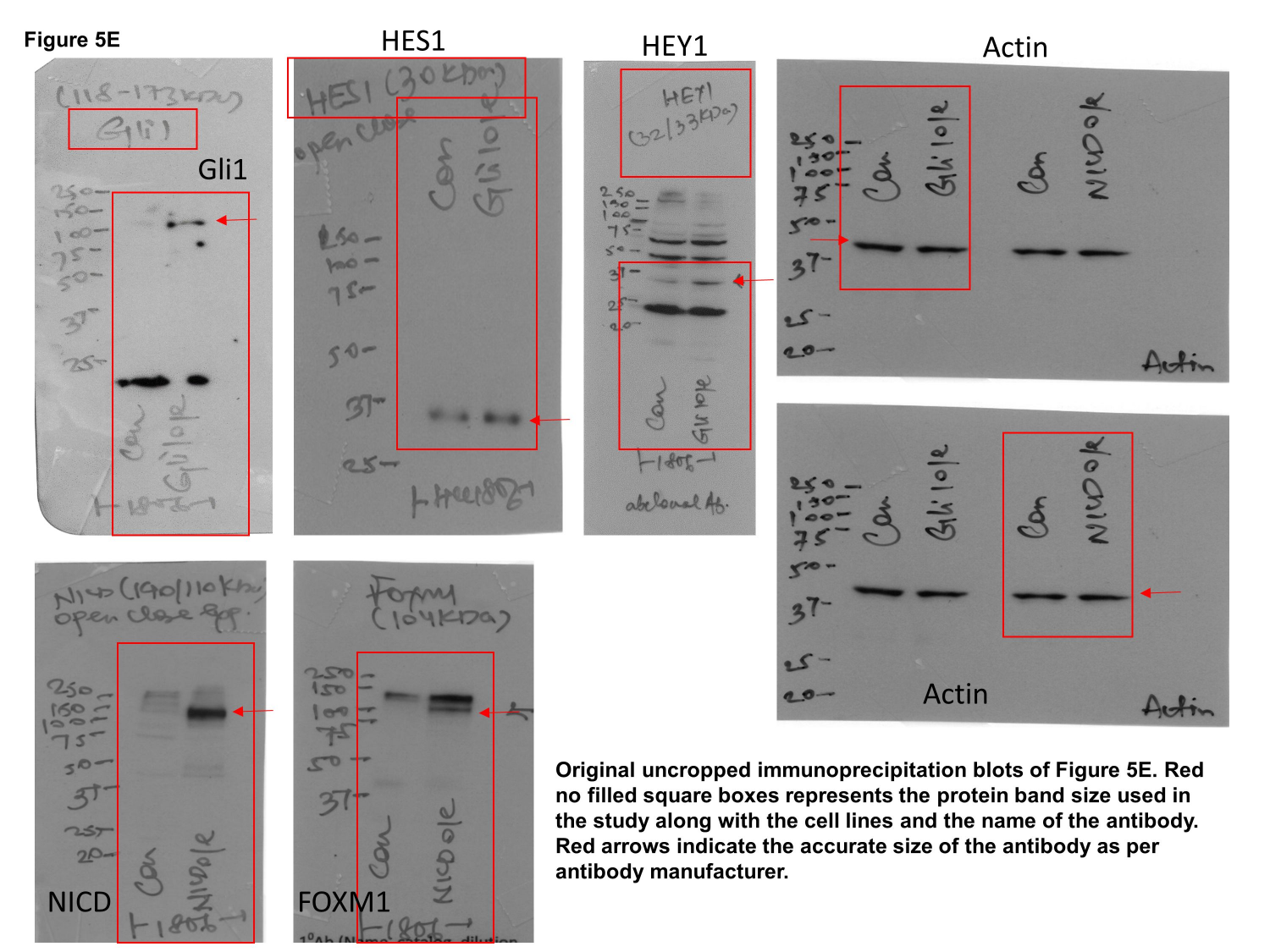

Supplement: Figure 5—source data 4. [file elife-70729-fig5-data4.pptx]
