# Supplementary material for: Concomitant activation of GLI1 and Notch1 contributes to racial disparity of human triple negative breast cancer progression
Source: eLife. 2021 Dec 10;10:e70729. doi: 10.7554/eLife.70729 (PMC8664295; doi:10.7554/eLife.70729)
Supplement: Transparent reporting form [file elife-70729-transrepform1.pdf]

Westbrook Centre
Milton Road
Cambridge CB4 1YG, UK

P 01223 855340
W elifesciences.org
T @elife

eLife's transparent reporting form

We encourage authors to provide detailed information *within their submission* to facilitate the interpretation and replication of experiments. Authors can upload supporting documentation to indicate the use of appropriate reporting guidelines for health-related research (see EQUATOR Network), life science research (see the BioSharing Information Resource), or the ARRIVE guidelines for reporting work involving animal research. Where applicable, authors should refer to any relevant reporting standards documents in this form.

If you have any questions, please consult our Journal Policies and/or contact us: editorial@elifesciences.org.

Sample-size estimation

- You should state whether an appropriate sample size was computed when the study was being designed
- You should state the statistical method of sample size computation and any required assumptions
- If no explicit power analysis was used, you should describe how you decided what sample (replicate) size (number) to use

Please outline where this information can be found within the submission (e.g., sections or figure legends), or explain why this information doesn't apply to your submission:

All the experiments were conducted in triplicates. 5-6 mice/group were used for each treatment group. This information is available in the Materials and Methods section.

Replicates

- You should report how often each experiment was performed
- You should include a definition of biological versus technical replication
- The data obtained should be provided and sufficient information should be provided to indicate the number of independent biological and/or technical replicates
- If you encountered any outliers, you should describe how these were handled
- Criteria for exclusion/inclusion of data should be clearly stated
- High-throughput sequence data should be uploaded before submission, with a private link for reviewers provided (these are available from both GEO and ArrayExpress)

Please outline where this information can be found within the submission (e.g., sections or figure legends), or explain why this information doesn't apply to your submission:

All the experiments were conducted thrice (biological replicates) in triplicates (technical replicates).

eLife Sciences Publications, Ltd is a limited liability non-profit non-stock corporation incorporated in the State of Delaware, USA, with company number 5030732, and is registered in the UK with company number FC030576 and branch number BR015634 at the address Westbrook Centre, Milton Road, Cambridge CB4 1YG.

Statistical reporting

- Statistical analysis methods should be described and justified
- Raw data should be presented in figures whenever informative to do so (typically when N per group is less than 10)
- For each experiment, you should identify the statistical tests used, exact values of N, definitions of center, methods of multiple test correction, and dispersion and precision measures (e.g., mean, median, SD, SEM, confidence intervals; and, for the major substantive results, a measure of effect size (e.g., Pearson's r, Cohen's d)
- Report exact p-values wherever possible alongside the summary statistics and 95% confidence intervals. These should be reported for all key questions and not only when the p-value is less than 0.05.

Please outline where this information can be found within the submission (e.g., sections or figure legends), or explain why this information doesn't apply to your submission:

> Information about the statistical analysis is included in the Material and Methods section. P-values are reported within the figure legends. All the experiments were conducted in triplicates. 5-6 mice/group were used for each treatment group.
>
> Statistical analyses were exploratory. Analyses were done using R and GraphPad Prism 5. Data was transformed when necessary to achieve normality. One-way ANOVA analysis was conducted for overall comparison across multiple groups. Pairwise comparisons were done using Student's t-test with Bonferroni correction. Kaplan-Meier survival curves were reported for survival outcomes and log-rank test was conducted to compare survival outcomes between groups. Results were considered to be significant if p-value < 0.05.

(For large datasets, or papers with a very large number of statistical tests, you may upload a single table file with tests, Ns, etc., with reference to sections in the manuscript.)

Group allocation

- Indicate how samples were allocated into experimental groups (in the case of clinical studies, please specify allocation to treatment method); if randomization was used, please also state if restricted randomization was applied
- Indicate if masking was used during group allocation, data collection and/or data analysis

Please outline where this information can be found within the submission (e.g., sections or figure legends), or explain why this information doesn't apply to your submission:

> We utilized a Tissue microarray consisting of 25 AA and 46 WA TNBC specimens that was generated at Yale Developmental Histology Lab using TNBC specimens that were diagnosed at the Yale-New Haven Hospital, Connecticut, USA between 1996 and 2004. TNBC status (absence of ER, PR, and HER2) was determined by IHC at the Yale Developmental Histology Lab.

Additional data files ("source data")

- We encourage you to upload relevant additional data files, such as numerical data that are represented as a graph in a figure, or as a summary table
- Where provided, these should be in the most useful format, and they can be uploaded as "Source data" files linked to a main figure or table
- Include model definition files including the full list of parameters used

eLife Sciences Publications, Ltd is a limited liability non-profit non-stock corporation incorporated in the State of Delaware, USA, with company number 5030732, and is registered in the UK with company number FC030576 and branch number BR015634 at the address Westbrook Centre, Milton Road, Cambridge CB4 1YG.

- Include code used for data analysis (e.g., R, MatLab)
- Avoid stating that data files are "available upon request"

Please indicate the figures or tables for which source data files have been provided:

We have included the source data for all the figures.
